# Atovaquone-Proguanil: A Promising Therapy for Persistent Relapsing Babesiosis

**DOI:** 10.1155/2024/7168928

**Published:** 2024-05-14

**Authors:** Mahum Shahid, Brendan Wechsler, Vinod Parameswaran, Mark Anthony Diaz

**Affiliations:** ^1^University of South Dakota Sanford School of Medicine, Vermillion, SD, USA; ^2^University of Nebraska Medical Center, Department of Radiology, Omaha, NE, USA; ^3^Avera Medical Group, Oncology and Hematology Department, Sioux Falls, SD, USA

## Abstract

We report a challenging case of persistent relapsing babesiosis in an immunocompromised host that was successfully managed with atovaquone-proguanil (Malarone). Malignant B-cell transformation and immunosuppressants, such as rituximab, deplete normal B-cells which normally produce antibodies to combat Babesia infection. Treatment can be prolonged and challenging in immunocompromised hosts. Atovaquone-proguanil (Malarone) is a novel therapy that can be used as part of a salvage regimen in case antimicrobial resistance or failure exists. Weighing the risks and benefits of continuing cancer therapy treatment or reducing the level of immunosuppression may aid in treatment. These are just as important as the choice of antimicrobial therapy for effective treatment and eradication of Babesia infection, especially in immunocompromised hosts.

## 1. Introduction

The northeastern and northern midwestern regions of the United States are endemic with babesiosis secondary to *B. microti*, the primary species causing the majority of babesiosis infections in humans [1]. Most of the infections occur from late spring to early autumn when human exposure to ticks (vectors) and rodents (primary reservoirs) increase [2]. Reported cases of transmission of *B. microti* through blood transfusion have also contributed to the rising number of cases. Babesiosis has a variable presentation with clinical manifestations ranging from asymptomatic disease to severe disease more commonly observed in immunocompromised patients. Within this immunocompromised population, the fatality rate can be as high as 21% [3]. The early diagnosis and appropriate treatment regimen in such cases are just as important as reducing, if not eliminating, immunosuppression. In addition, this case adds to the growing evidence of atovaquone-proguanil (Malarone) as part of a regimen for treatment of babesiosis.

## 2. Case Presentation

The patient is a 73-year-old male with a history of chronic lymphocytic leukemia (CLL) in complete remission and well controlled on ibrutinib therapy for several years. He initially presented to his primary care provider with generalized weakness and easy fatigability and was found to have unexplained anemia. After several weeks, his symptoms were complicated by episodes of confusion and falls. He was subsequently referred to the hematology clinic for further workup where he was started on prednisone therapy and was eventually admitted to the hospital on 22 October 2020, for persistent symptoms.

He denied any episodes of fever, night sweats, chills, unwanted weight loss, rashes, focal weakness, or loose bowel movements. His hobbies included fishing and hunting, but he did not recall any specific instance of a tick or insect bite. He had multiple blood transfusions in the past for treatment of his anemia. He reported a recent trip to Long Island, New York, in August, but denied any known recent illnesses or exposure. He denied any travel outside of the United States, including to any malaria-endemic countries. He denied consumption of game meat or unfiltered water. The patient had no history of tattoos or any sexually transmitted disease. He recalled a remote history of hemolysis associated with quinine use.

### 2.1. Investigations

**Figure 1:**
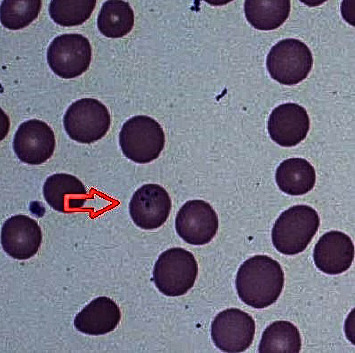
Peripheral smear of the patient showing the pathognomonic “Maltese Cross” tetrad form within an infected red blood cell.

His physical examination was unremarkable other than dyspnea after prolonged conversation. His laboratory work on this admission noted unexplained anemia with a hemoglobin of 5.1 g/dL and hematocrit of 15.7 before receiving several blood transfusions, as well as total bilirubin of 5.1 mg/dL, lactate dehydrogenase of 1699 U/L, and a peripheral smear showing rare spherocytes suggestive of a hemolytic process. Ibrutinib was discontinued; steroids with intravenous immunoglobulins (IVIGs) and rituximab were started for suspected CLL-associated Coombs-negative hemolytic anemia or acute viral illness. His symptoms mildly improved, and he was discharged on 1 November 2019. Within several weeks, he developed severe progressive fatigability and shortness of breath that warranted repeat hospitalization on 31 December 2019. CBC noted WBC of 14.3 K/*μ*L, Hgb of 7.9 g/dL, and platelets of 68 K/*μ*L. A bone marrow biopsy was performed which noted hemophagocytosis. Initial review of the peripheral blood smear showed ring forms suggestive of a parasitic infection suspicious for malaria. A closer look at the slides with the pathologist revealed tetrad forms within RBCs resembling “Maltese crosses” (Figure 1).

### 2.2. Treatment

He was initially treated with atovaquone and azithromycin for 4 weeks. Peripheral smears were negative on 9 January 2020, after about one week of therapy. The follow-up of tick bone disease antibody panel was consistent with *Babesia microti* infection, IgM antibody titer of 1 : 40 (greater than 1 : 20 is positive), and negative for Anaplasma, Ehrlichia, and Lyme disease. He was discharged on 24 January 2020, following resolution of symptoms and proven clearance of parasitemia.

**Figure 2:**
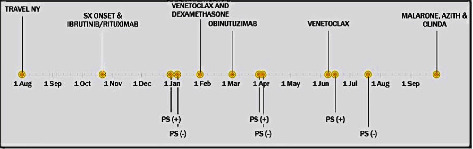
Patient's timeline from day of travel until final treatment with remission. (PS: peripheral smear status).

On 30 March 2020, just 8 weeks after completion of treatment, he presented once again with symptoms of severe generalized weakness and easy fatigability. Peripheral smear was consistent with relapsed babesiosis. A new regimen of azithromycin, atovaquone, and clindamycin combination was initiated. The follow-up peripheral smear once again cleared after a few days and the new regimen was continued for 8 weeks. On 16 June 2020, his anticipated last week of therapy, and he once again presented with the same symptoms of generalized weakness and peripheral smear again showed relapsing babesiosis. To treat this relapsing infection, he was started on a combination of atovaquone-proguanil (Malarone), azithromycin, and clindamycin. The review of his outpatient medications noted treatments with ibrutinib, venetoclax, and dexamethasone two weeks prior to his symptoms and subsequent relapsing babesiosis. At this time, chemotherapy treatment for his CLL was withheld after considering risks and benefits. The parasitemia was proven cleared on 20 July 2020, after 4 weeks; a total treatment duration of 8 weeks was administered to ensure infection clearance. The timeline of treatment selection and medication adjustments is outlined in (Figure 2). Continual follow-up peripheral smears for the next year demonstrated no relapse of babesiosis.

## 3. Discussion

Babesia is mostly transmitted by an infected tick bite and other reported modes of transmission include blood transfusion, vertical transmission, and organ transplantation. Small rodents serve as the primary host of babesiosis, whereas *Ixodes scapularis* is the primary tick vector responsible for transmission [3]. Babesiosis has a variable presentation with clinical manifestations ranging from asymptomatic disease to severe symptoms, resulting in death. Fatigue and malaise are the initial symptoms after an incubation period of 1–6 weeks (from tick bite) or up to 6 months (from blood transfusion). Patients subsequently develop fevers, chills, myalgia, anorexia, and headaches. Common laboratory findings include hemolytic anemia, thrombocytopenia, elevated transaminases, and creatinine levels.

Relapsing babesiosis despite standard antimicrobial treatment has been observed in immunocompromised patients. Any immunocompromised patient with similar signs and symptoms discussed above, recent travel to an endemic area, or history of a blood transfusion in the last 6 months should have babesiosis in the list of differential diagnoses. Confirmation of diagnosis shows parasites within the red blood cells (RBCs) on a thin slide with Giemsa or Wright stain. The tetrad of merozoites (Maltese-cross appearance) within an RBC is considered pathognomonic for babesiosis. Alternatively, a real-time polymerase chain reaction (PCR) assay is more sensitive than blood smear and detects as few as 10 parasites per microliter of blood [4].

A weakened immune system or medications that affect the B-cell activity can favor persistence of the infection in the immunocompromised host, as seen in our patient. The clearance of Babesia from an infected host includes the spleen and activated plasma cells, derived from B cells, which secrete antibodies to decrease parasite entry into erythrocytes and increase their opsonization by macrophages. They also enhance the activity of natural killer cells via antibody-dependent cellular cytotoxicity and complement activation. The antibodies produced by plasma cells can bind to these adherence proteins and block their function. Babesia also produces adhesion molecules which allow adherence of parasite-infected erythrocytes to the vascular endothelium, thereby allowing completion of their life cycle without exposure to the splenic immune system [5].

Therapies commonly utilized for the treatment of malignancy, solid or hematological, can weaken the immune system and put the patient at risk for developing persistent infections. Lymphoma and lymphoblastic leukemia therapies are specifically designed to target neoplastic cells of lymphoid origin and can target the normal lymphocyte cells as well. Bruton tyrosine kinase (BTK) inhibitors (ibrutinib and acalabrutinib), BCL-2 inhibitor (venetoclax), and monoclonal antibodies that target B-cell CD20 receptors (rituximab and obinutuzumab) are the drug class therapies commonly used to treat symptomatic or advanced CLL as well as other lymphoid malignancies [6]. They affect the activity of healthy B-cells during the duration of treatment and exert their effect on the immune system even after cessation of the drug. It was seen that B-cell depletion was sustained for up to 6–9 months and B-cell recovery began at 6 months following completion of treatment; median B-cell levels returned to normal by 12 months following completion of treatment, with full gain of function at 18 months [7]. Our patient received several therapies from each of the above-described classes of drugs that affect the activity of B cells and, therefore, was unable to clear the infection despite adequate antibiotic coverage. It was only after all B-cell-targeted therapy was stopped that our patient achieved remission.

Disease is noted to be more severe with frequent relapses in immunocompromised patients including patients with HIV/AIDS, asplenia, malignancy, and patients receiving immunosuppressant medications. In general, treatment consists of a combination of antimicrobial therapy that includes atovaquone and azithromycin as first line therapy. Treatment of 7–10 days is usually effective with eradication of the parasites. Second-line antimicrobial regimen includes clindamycin and quinine, which is the standard of care for severely ill patients as per the CDC and IDSA guidelines [4]. Response to these drugs in immunocompetent patients is generally favorable [8].

However, management is more challenging in immunocompromised hosts as prolonged parasitemia and relapses are more common. There are no standard treatment guidelines for this cohort of patients with a weakened immune system and persistent parasitemia. Duration of therapy is usually extended for at least 6 weeks, which includes at least 2 weeks of treatment after negative blood smears. Exchange transfusion is also sometimes used for patients with significant hemolysis, severe end organ damage and a parasitemia of >10%.

Several studies have compared different drug regimens in patients with persistent relapsing babesiosis to postulate an effective regimen. Malarone, although rarely, has been used in relapsed patients, but data remain insufficient on an effective duration of treatment to propose it as one of the first-line agents. A retrospective case-control study compared 14 “case” patients, who had persistent Babesia infection with severe morbidity or mortality, with 46 “control” subjects who responded to a single course of standard therapy. The treatment, clinical prognosis, and immunological status of these 14 were compared with 46 patients who had a benign course with infection clearance with one course of antibiotics. It was observed that all the case patients were immunosuppressed when they contracted the infection whereas less than 10% of the control subjects had immunosuppression. The case patients experienced severe symptoms and 3 of the patients eventually passed away despite treatment [7]. Although several combinations of antibiotics were used in these patients, only 2 of the case patients had received atovaquone-proguanil combined with other antibiotics and the combination was not found to be curative in any of the patients. Another case describes the successful use of Malarone in treating a patient with acquired immune deficiency syndrome (AIDS) who failed to respond to the first-line treatment therapy for babesiosis [9].

One additional case series of three immunocompromised patients showed more severe disease and resistance to atovaquone-azithromycin combination therapy. The study described resistance as microbiological relapse despite 28 days of uninterrupted treatment. All three patients had history of B-cell lymphomas and splenectomies. Two of the patients also had rituximab therapy and one patient had a solid organ transplant. Interestingly, one of these patients received the same regimen as our patient, a combination of Malarone, azithromycin, and clindamycin, for 13 months [4].

Our patient was immunocompromised at the baseline given his CLL and received further immunosuppression with BCL2/BTK inhibition, steroids, and anti-CD20 therapy that may explain the prolonged and relapsing Babesia infection. Our case highlights the importance of choosing the appropriate antimicrobial therapy as it may be just as important as controlling the other risk factors for infection, in addition to discussing risks and benefits of withholding immunosuppressive agents. Moreover, it adds to the growing evidence of the use of Malarone as a treatment option to treat persistent relapsing babesiosis in immunocompromised hosts.

### 3.1. Limitations

The limitations of this case report study include the sample size of one patient despite close monitoring and follow-up of the patient's outcome with treatment of underlying infection. An improvement in future studies may include a larger patient population for statistical analysis for treatment of resistant babesiosis in immunocompromised hosts with a drug regimen including Malarone.

## Data Availability

No data were used to support the findings of this study.
